# E-Selectin, ICAM-1, and ET-1 Biomarkers Address the Concern of the Challenging Diagnosis of Interstitial Lung Disease in Patients with Autoimmune Diseases

**DOI:** 10.3390/ijms241512518

**Published:** 2023-08-07

**Authors:** Verónica Pulito-Cueto, Sara Remuzgo-Martínez, Fernanda Genre, Belén Atienza-Mateo, Víctor M. Mora-Cuesta, David Iturbe-Fernández, Leticia Lera-Gómez, María Sebastián Mora-Gil, Virginia Portilla, Alfonso Corrales, Ricardo Blanco, José M. Cifrián, Miguel A. González-Gay, Raquel López-Mejías

**Affiliations:** 1 Immunopathology Group, Marqués de Valdecilla University Hospital-Marqués de Valdecilla Research Institute (IDIVAL), 39011 Santander, Spain; mateoatienzabelen@gmail.com (B.A.-M.); victormanuel.mora@scsalud.es (V.M.M.-C.); diturfer@gmail.com (D.I.-F.); msebastian@idival.org (M.S.M.-G.); virgiportilla@hotmail.com (V.P.); afcorralesm@hotmail.com (A.C.); ricardo.blanco@scsalud.es (R.B.); josemanuel.cifrian@scsalud.es (J.M.C.); rlopezmejias78@gmail.com (R.L.-M.); 2 Department of Rheumatology, Hospital Universitario Marqués de Valdecilla, 39008 Santander, Spain; 3 Marqués de Valdecilla Research Institute (IDIVAL), 39011 Santander, Spain; sara.r.mtz@gmail.com (S.R.-M.); fernandagenre@gmail.com (F.G.); miguelaggay@hotmail.com (M.A.G.-G.); 4 Department of Pneumology, Hospital Universitario Marqués de Valdecilla, 39008 Santander, Spain; 5 Department of Microbiology, Hospital Universitario Marqués de Valdecilla, 39008 Santander, Spain; letizialera@hotmail.com; 6 School of Medicine, Universidad de Cantabria, 39011 Santander, Spain; 7 Department of Rheumatology, IIS-Fundación Jiménez Díaz, 28040 Madrid, Spain

**Keywords:** E-selectin, ICAM-1, ET-1, interstitial lung disease, autoimmune diseases, rheumatoid arthritis, systemic sclerosis, biomarkers, pulmonary fibrosis

## Abstract

Interstitial lung disease (ILD) constitutes the most critical comorbidity in autoimmune diseases (ADs) and its early diagnosis remains a challenge for clinicians. Accordingly, we evaluated whether E-selectin, ICAM-1, and ET-1, key molecules in endothelial damage, could be useful biomarkers for the detection of AD-ILD^+^. We recruited patients with rheumatoid arthritis (RA)-ILD^+^ (*n* = 21) and systemic sclerosis (SSc)-ILD^+^ (*n* = 21). We included comparison groups of patients: RA-ILD^−^ (*n* = 25), SSc-ILD^−^ (*n* = 20), and idiopathic pulmonary fibrosis (IPF) (*n* = 21). Serum levels of these proteins were determined by ELISA. E-selectin, ICAM-1, and ET-1 serum levels were increased in RA-ILD^+^ and IPF patients in comparison to RA-ILD^−^ patients. Additionally, SSc-ILD^+^ and IPF patients exhibited higher ICAM-1 levels than those with SSc-ILD^−^. The ability of E-selectin, ICAM-1, and ET-1 to discriminate RA-ILD^+^ from RA-ILD^−^ patients, and ICAM-1 to distinguish SSc-ILD^+^ from SSc-ILD^−^ patients was confirmed using ROC curve analysis. Furthermore, elevated levels of ET-1 and E-selectin correlated with lung function decline in RA-ILD^+^ and SSc-ILD^+^ patients, respectively. In conclusion, our findings support the relevant role of E-selectin, ICAM-1, and ET-1 in RA-ILD^+^ patients as well as of ICAM-1 in SSc-ILD^+^ patients, constituting potential screening blood biomarkers of ILD in AD. Moreover, this study suggests ET-1 and E-selectin as possible indicators of worsening lung function in RA-ILD^+^ and SSc-ILD^+^ patients, respectively.

## 1. Introduction

Vascular and immunologic processes are central to the pathogenesis of autoimmune diseases (ADs) [1,2,3]. Patients with ADs present increased activation of immune cells, which can infiltrate into internal organs promoting vascular damage [4,5]. Thus, the prognosis and outcome of ADs are partially dependent on these pathologic conditions taking place in different organs, with the lung being one of the main organs affected [5,6,7,8,9,10]. In particular, AD patients with interstitial lung disease (ILD), a lung parenchymal disorder with great prevalence in patients with rheumatoid arthritis (RA) and systemic sclerosis (SSc), suffer from higher disease-related morbidity and mortality, meaning that ILD is one of the most serious complications in AD patients [5,6,7,8,9,10]. Therefore, predicting the chance of AD patients who progress to a severe state of the disease developing ILD is particularly important. Although, currently, the evaluation of lung fibrosis in AD patients primarily relies on pulmonary function tests (PFTs) and high-resolution computed tomography (HRCT), the early and precise diagnosis of AD-ILD^+^ often remains a challenge [5,6,7,8,9]. Additionally, the diagnosis complexity of AD-ILD^+^ increases due to the similarity of clinical, pathological, and epidemiological features with idiopathic pulmonary fibrosis (IPF) [7,8,9]. Of note, many studies of biomarkers for the risk of developing ILD are underway, but only two protein candidates deserve special attention—Krebs von den Lungen and surfactant—and these have been studied in AD-ILD^+^ with promising results. However, currently, no individual or group serum biomarkers have been identified as sufficiently robust biomarkers to justify their use in clinical practice [5,11].

Cellular-level changes that lead to ILD development likely begin years before the onset of symptoms. This has led to subclinical ILD research gaining interest in recent years as a way to identify novel risk factors and early mechanisms of lung fibrosis [5,12]. Specifically, given that lung vasculopathy is one of the necessary steps in the development of lung fibrosis and subsequent ILD, attention has been increasingly focused on endothelial activation [3,4,5,12,13,14,15]. In fact, we have previously proposed that a variety of biomarkers of endothelial activation could play a major role in the pathogenesis of AD-ILD^+^ [16,17,18,19,20].

Pulmonary endothelial damage and fibrosis implicate the cellular infiltration in the lung parenchyma [4,5,10,12], an action mainly mediated by the production of soluble mediators. Leukocyte recruitment is generally achieved through cell adhesion molecules, with the two major molecules being E-selectin and intercellular adhesion molecule-1 (ICAM-1). Moreover, another essential trigger of endothelial damage is endothelin-1 (ET-1), the most potent vasoconstrictor [2,13,14,15,21,22,23]. Alteration of these molecules is associated with endothelial dysfunction, a pathological condition thought to represent a key process in the initial step of the AD pathogenesis [1,2]. The importance of E-selectin, ICAM-1, and ET-1 is such that an expanding body of evidence demonstrates that increased levels of these molecules are associated with a wide variety of inflammatory and immune-mediated mechanisms, with the molecules being described as biomarkers of endothelial dysfunction in several disorders such as RA, SSc, systemic lupus erythematosus, spondyloarthropathies, psoriasis, and cerebral small vessel disease [1,2,24,25].

Accordingly, we hypothesized that these endothelial-damage-related molecules may be linked to underlying biological processes that may be ongoing in AD, and that the molecules would change the clinical features of these patients over time leading to the development of ILD. Based on this, and the lack of literature pertaining to circulating endothelial-damage-related molecules in this context, we sought to determine if E-selectin, ICAM-1, and ET-1 could be useful as screening biomarkers for the detection of AD-ILD^+^. 

## 2. Results

### 2.1. E-Selectin, ICAM-1, and ET-1 as Biomarkers for the Presence of ILD in RA Patients

**Figure 1:**
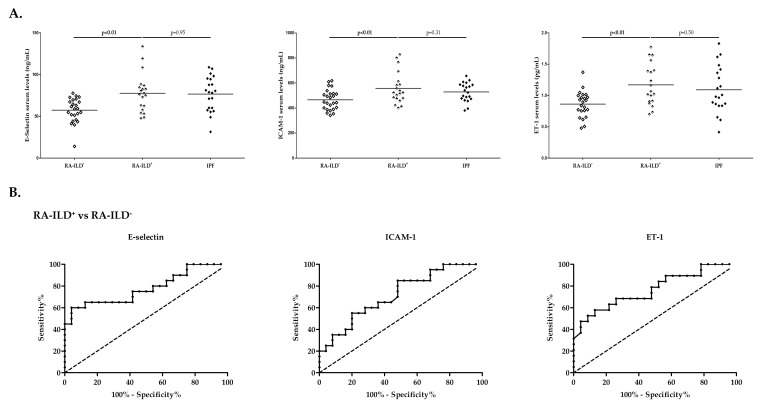
Roles of E-selectin, ICAM-1, and ET-1 in RA-ILD^+^. Differences in serum levels of E-selectin, ICAM-1, and ET-1 between patients with RA-ILD^+^ and those with RA-ILD^−^ and IPF (**A**) and ROC curve analysis for the discrimination of RA-ILD^+^ from RA-ILD^−^ (**B**). ICAM-1: intercellular adhesion molecule 1; ET-1: endothelin 1; RA: rheumatoid arthritis; ILD: interstitial lung disease; IPF: idiopathic pulmonary fibrosis. Significant results are highlighted in **bold**.

RA-ILD^+^ patients showed increased levels of E-selectin, ICAM-1, and ET-1 compared to those with RA-ILD^−^ (*p* < 0.01 in all the cases, Figure 1A, Table S1 (see Additional File S1)). Interestingly, the ability of serum E-selectin, ICAM-1, and ET-1 levels to discriminate patients with RA-ILD^+^ from those with RA-ILD^−^ was confirmed by performing ROC curve analysis (AUC: 0.78, *p* < 0.01; AUC: 0.72, *p* = 0.01; AUC: 0.77, *p* < 0.01, respectively, Figure 1B, Table 1). The optimal cutoff value for E-selectin, ICAM-1, and ET-1 revealed that the best sensitivity and specificity was 74.56 ng/mL, 451.70 ng/mL, and 1.02 pg/mL, respectively (Table 1).

Moreover, patients with RA-ILD^+^ presented similar levels of E-selectin, ICAM-1, and ET-1 to those with IPF (Figure 1A, Table S1 (see Additional File S1)). 

### 2.2. ICAM-1 Implicated in the Presence of ILD in SSc Patients 

**Figure 2:**
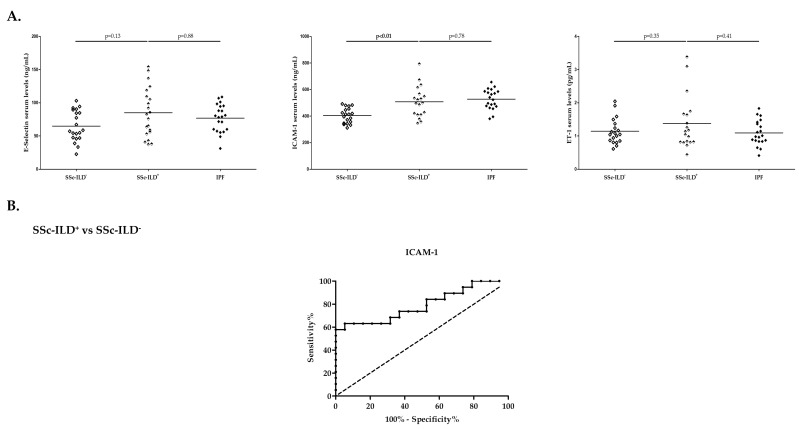
Roles of E-selectin, ICAM-1, and ET-1 in SSc-ILD^+^. Differences in serum levels of E-selectin, ICAM-1, and ET-1 between patients with SSc-ILD^+^ and those with SSc-ILD^−^ and IPF (**A**) and ROC curve analysis of ICAM-1 for the discrimination of SSc-ILD^+^ from SSc-ILD^−^ (**B**). ICAM-1: intercellular adhesion molecule 1; ET-1: endothelin 1; SSc: systemic sclerosis; ILD: interstitial lung disease; IPF: idiopathic pulmonary fibrosis. Significant results are highlighted in **bold**.

Higher ICAM-1 serum levels were found in patients with SSc-ILD^+^ compared to those with SSc-ILD^−^ (*p* < 0.01, Figure 2A, Table S1 (see Additional File S1)), although no significant differences were observed between these patients when E-selectin and ET-1 were evaluated (Figure 2A, Table S1 (see Additional File S1)). Of note, the ROC curve supported the utility of ICAM-1 for the differentiation of patients with SSc-ILD^+^ from those with SSc-ILD^−^ (Figure 2B, Table 1). The AUC was 0.79 (*p* < 0.01) and the optimal cutoff value was 484.70 ng/mL (Figure 2B, Table 1).

Additionally, no differences in levels of E-selectin, ICAM-1, and ET-1 were found between patients with SSc-ILD^+^ and those with IPF (Figure 2A, Table S1 (see Additional File S1)). 

### 2.3. Association of E-Selectin, ICAM-1, and ET-1 with Clinical Characteristics of Patients with RA-ILD^+^ and SSc-ILD^+^

**Figure 3:**
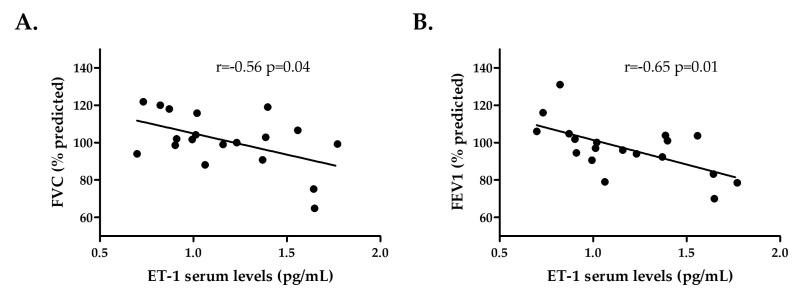
Relationship of ET-1 serum levels with FVC (**A**) and FEV1 (**B**) in patients with RA-ILD^+^. ET-1: endothelin 1; FVC: forced vital capacity; FEV1: forced expiratory volume in one second; RA: rheumatoid arthritis; ILD: interstitial lung disease.

A negative correlation between ET-1 serum levels and both FVC and FEV1 was noticed in patients with RA-ILD^+^ (r = −0.56, *p* = 0.04 and r = −0.65, *p* = 0.01, respectively, Figure 3, Table 2). 

**Figure 4:**
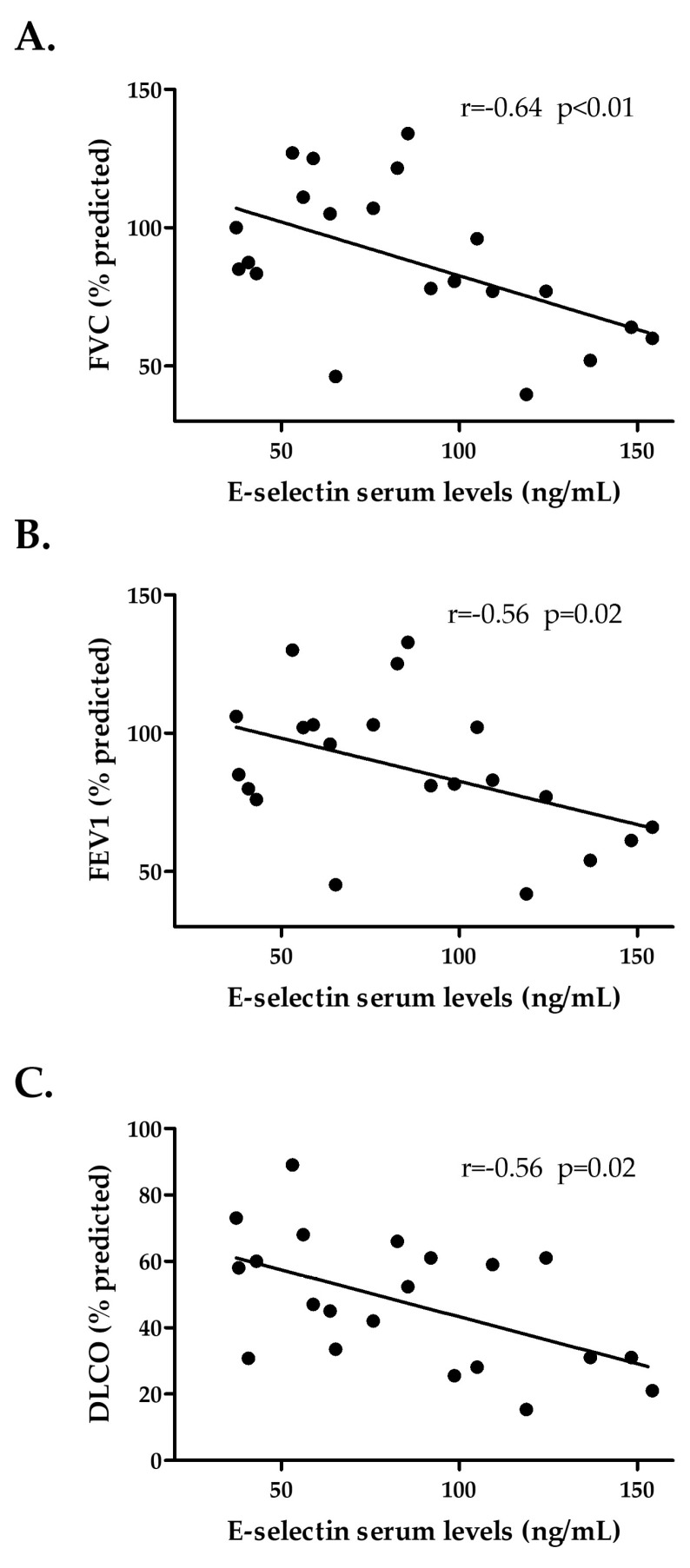
Relationship of E-selectin serum levels with FVC (**A**), FEV1 (**B**), and DLCO (**C**) in patients with SSc-ILD^+^. FVC: forced vital capacity; FEV1: forced expiratory volume in one second; DLCO: diffusing capacity of the lung for carbon monoxide; SSc: systemic sclerosis; ILD: interstitial lung disease.

Furthermore, E-selectin serum levels were negatively correlated with FVC, FEV1, and DLCO in patients with SSc-ILD^+^ (r = −0.64, *p* < 0.01; r = −0.56, *p* = 0.02; and r = −0.56, *p* = 0.02, respectively, Figure 4A–C, Table 3). 

No relationships between E-selectin, ICAM-1, and ET-1 levels and other clinical characteristics of patients with RA-ILD^+^ and SSc-ILD^+^ were observed (Table 2 and Table 3). 

## 3. Discussion

The development, progression, and severity of AD are partially determined by vascular damage, a pathological process that can influence several organs of AD patients, with the lung being one of the main organs affected. Specifically, ILD constitutes the most critical comorbidity in AD; therefore, its early diagnosis is pivotal to avoiding irreversible lung damage in these patients [4,5,6,7,8,9,10]. However, no definitive serum biomarkers are available to identify ILD in AD patients, denoting the need for novel biomarkers that address this concern. Accordingly, we evaluated whether a single measure of soluble E-selectin, ICAM-1, or ET-1 is useful as a screening biomarker for the detection of AD-ILD^+^.

This work evidenced an increase of E-selectin, ICAM-1, and ET-1 circulating levels in patients with RA who exhibited ILD. These findings are in line with the theory of the pathogenesis of ILD in AD. Thereby, it is plausible to think that soluble E-selectin, ICAM-1, and ET-1 are produced by endothelial cells upon stimulation by proinflammatory cytokines, increasing their circulating levels. This systemic inflammatory process could be causing the entry of inflammatory cells into the interstitial and alveolar space, generating endothelial and epithelial damage, and promoting the recruitment and activation of lung fibroblasts and myofibroblasts, key drivers of fibrosis. These cellular changes lead to overproduction and abnormal deposition of extracellular matrix triggering the scarring of lung tissue and pulmonary fibrosis [5,9,10,11,26]. Hence, our results may point to a role for E-selectin, ICAM-1, and ET-1 as serum biomarkers of endothelial injury related to fibrotic processes that occur in the lung of RA patients. 

Another result that deserves consideration is the similar concentrations of E-selectin, ICAM-1, and ET-1 in RA-ILD^+^ and IPF patients, a finding that reveals a special contribution of lung vasculopathy in the alteration of these molecules. Specifically, the highest E-selectin levels in patients with RA-ILD^+^ and IPF could suggest that leukocyte-mediated lung injury is necessary for the initiation and/or propagation of the fibrogenic process. Thus, it has previously been considered a relevant protein in the progression of pulmonary fibrosis [12,15,27,28,29]. Besides, it is comprehensible to suggest that enhanced levels of ICAM-1 in patients with ILD, regardless of whether they have RA-ILD^+^ or IPF, could be due to a compensatory response to lung damage. In this regard, other organs release ICAM-1 to the circulation because of the loss of the alveolar-capillary units, the main source of its production [12,29]. In addition, its role in lung involvement could be linked to its ability to regulate the accumulation of profibrotic cells in the lungs [30]. Concerning ET-1, hypoxia could be stimulating the production of this protein in patients with RA-ILD^+^ and IPF, since it is one of its most important inducers [31]. On the other hand, its profibrotic role in these patients could also be a consequence of its ability to stimulate fibroblast proliferation and activation, as well as its effects on alveolar epithelial cell apoptosis [13,31,32]. In line with this idea, and our results, previous studies have described a role for ET-1 in the development of pulmonary fibrosis [13,31,32,33,34]. It is noteworthy that our study exhibited that during lung injury and fibrosis in RA patients, increased serum levels of ET-1 correlated with lung-function decline, suggesting that circulating ET-1 measured may also predict worsening of lung function in these RA-ILD^+^ patients. 

Interestingly, our study also showed enhanced ICAM-1 levels in patients with SSc-ILD^+^ and IPF in relation to those with SSc-ILD^−^, evidencing a potential influence of lung vasculopathy on circulating ICAM-1. Thus, our data point to ICAM-1 as a possible indicator of the presence of ILD in patients with SSc. The higher levels of ICAM-1 in SSc-ILD^+^ patients makes sense considering that it is essential for leukocyte diapedesis, being previously described as a contributor to the development of inflammation and fibrosis in SSc via induction of the infiltration and activation of leukocytes [35]. According to our results, ICAM-1 has been considered a marker of respiratory dysfunction showing higher concentrations in SSc patients with lung involvement [3,35,36,37,38,39].

However, RA-ILD^+^ and SS-ILD^+^ are diseases with completely different final phenotypes, so it would not be unexpected to find different patterns of molecular expression in the blood of patients with these diseases. In this sense, we observed similar serum E-selectin and ET-1 levels in all patients with SSc, regardless of the presence of ILD. Nevertheless, it is important to mention that E-selectin is somehow related to ILD in SSc since it was specifically correlated with worse respiratory function in patients with SSc-ILD^+^. In agreement with our results, E-selectin was previously associated with lower FVC and respiratory mortality of subclinical ILD [12]. Therefore, our findings suggest that serum E-selectin is useful in predicting subsequent respiratory dysfunction in patients with SSc-ILD^+^.

Briefly, the endothelial activation phenomenon observed in the serum of ILD patients specifically, could be reflecting the pulmonary endothelium activation that occurs by the presence of interstitial lung lesions in AD patients, offering important insights into the biologic processes subjacent to AD-ILD^+^ development. Hence, our work provides a way of finding novel biomarkers and therapeutic targets for AD-ILD^+^, and is helpful in providing guidance on the research of the pathogenesis of the disease.

## 4. Materials and Methods

### 4.1. Study Population

The study objective groups encompassed 21 patients with RA-ILD^+^ and 21 patients with SSc-ILD^+^. Furthermore, we included three comparative groups of patients: 25 with RA-ILD^−^, 20 with SSc-ILD^−^, and 21 with IPF. All these individuals were recruited from the Pneumology and Rheumatology departments of Hospital Universitario Marqués de Valdecilla (Santander, Spain), and peripheral venous blood samples were collected from all of them. 

RA patients met the 2010 American College of Rheumatology/European League Against Rheumatism (ACR/EULAR) criteria [40] and SSc patients fulfilled the 2013 ACR/EULAR criteria [41].

Pulmonary involvement was evaluated in all the patients by HRCT images of the chest and PFTs. Patients with each AD were classified by the presence or absence of ILD according to the American Thoracic and European Respiratory Society’s (ATS/ERS) criteria for ILD [42]. IPF patients met the ATS/ERS criteria [42]. 

Demographic and clinical features including sex, age, smoking history, C-reactive protein, erythrocyte sedimentation rate, antibody status, PFTs, and HRCT patterns were collected from most of the patients. In particular, HRCT patterns of ILD patients were stratified according to the criteria for the usual interstitial pneumonia (UIP) pattern of the Fleischner Society [43] (Table 4). 

All the experiments involving humans and human blood samples were carried out in accordance with the approved guidelines and regulations, according to the Declaration of Helsinki. All experimental protocols were approved by the Ethics Committee of clinical research of Cantabria, Spain (2016.092). All subjects gave written informed consent to participate in this study before their inclusion.

### 4.2. Determination of E-Selectin, ICAM-1, and ET-1 Serum Levels 

Serum samples were collected by centrifugation at 4500 rpm for 10 min from the blood samples of the patients. Serum samples were stored at −80 °C until use. Samples were diluted 1/5 and 1/10 for the determination of E-selectin and ICAM-1, respectively, while sample were not diluted for the analysis of ET-1. Serum levels of E-selectin, ICAM-1, and ET-1 were evaluated by a commercial enzyme-linked immunosorbent assay kit (E-selectin: BMS205, Invitrogen, Vienna, Austria; ICAM-1: BMS201, Invitrogen, Vienna, Austria; ET-1: DET100, R&D Systems, Minneapolis, MN, USA), in accordance with the manufacturers’ instructions. The optical density was quantified in a spectrophotometer (Multiskan FC, Thermo Scientific, Waltham, MA, USA) at wavelengths of 450 nm and 620 nm as reference. All the samples were analyzed in duplicate and quantified relative to a standard curve, using 5-parameter logistic regression for E-selectin and ICAM-1 and 4-parameter logistic regression for ET-1, as recommended by the manufacturer, through MyAssays^®^ Ltd 2021 online software.

### 4.3. Statistical Analyses

Continuous variables were expressed as mean ± standard deviation (SD) and categorical variables as number of individuals (*n*) and percentage (%). 

Analysis of variance (ANOVA) was used for the comparison of protein levels between the two study groups, adjusting for the following potential confounding factors: sex, age at the time of the study, and smoking history. When significant differences between groups were obtained, receiver operating characteristic (ROC) analysis was performed. The area under the curve (AUC) with a 95% confidence interval (CI) was calculated. The optimal cutoff values of E-selectin, ICAM-1, and ET-1 for discriminating AD-ILD+ from AD-ILD− were calculated by the Youden index (the higher value obtained from the formula sensitivity% + specificity%—100). 

The association of protein levels with continuous and categorical variables was analyzed via the estimation of Pearson’s partial correlation coefficient (r) and linear regression, respectively, adjusting for the above-mentioned potential confounding factors.

Statistically significant differences were considered as p < 0.05. Statistical analysis was performed using STATA statistical software 12/SE (Stata Corp., College Station, TX, USA). 

## 5. Conclusions

In conclusion, our findings suggest relevant roles of E-selectin, ICAM-1, and ET-1 in RA-ILD^+^ as well as of ICAM-1 in SSc-ILD^+^, supporting their utility as potential screening blood biomarkers of subclinical ILD in AD, contributing to the early diagnosis of the disease. Moreover, this study points to ET-1 and E-selectin as indicators of ongoing lung injury and the subsequent severity in RA-ILD^+^ and SSc-ILD^+^, respectively, helping to monitor disease progression and therapeutic responses.

## Figures and Tables

**Table 1:** ROC curve analysis for the discrimination of RA-ILD^+^ from RA-ILD^−^ and SSc-ILD^+^ from SSc-ILD^−^.

	AUC (95% CI)	*p*	Optimal Cutoff Value	Sensitivity (%)	Specificity (%)
**RA-ILD^+^ vs. RA-ILD^−^**					
E-selectin	0.78 (0.64–0.92)	<0.01	74.56 ng/mL	60.00	95.83
ICAM-1	0.72 (0.57–0.87)	0.01	451.70 ng/mL	85.00	52.00
ET-1	0.77 (0.62–0.91)	<0.01	1.02 pg/mL	57.89	86.96
**SSc-ILD^+^ vs. SSc-ILD^−^**					
ICAM-1	0.79 (0.65–0.94)	<0.01	484.70 ng/mL	63.2	94.7

ROC: receiver operating characteristic; RA: rheumatoid arthritis; ILD: interstitial lung disease; SSc: systemic sclerosis; AUC: area under the curve; CI: confidence interval; ICAM-1: intercellular adhesion molecule 1; ET-1: endothelin 1.

**Table 2:** Relationships of serum levels of E-selectin, ICAM-1, and ET-1 with clinical characteristics of RA-ILD^+^ patients.

	E-Selectin Serum Levels		ICAM-1 Serum Levels		ET-1 Serum Levels	
Variable	*r*	*p*	*r*	*p*	*r*	*p*
Duration of RA (years)	−0.42	0.10	−0.33	0.23	0.30	0.30
CRP (mg/dL)	−0.22	0.43	−0.17	0.56	0.36	0.23
ESR (mm/1st hour)	−0.08	0.77	0.17	0.55	0.07	0.82
FVC (% predicted)	0.00	0.99	−0.39	0.15	**−0.56**	**0.04**
FEV1 (% predicted)	0.05	0.87	−0.33	0.24	**−0.65**	**0.01**
DLCO (% predicted)	0.03	0.93	−0.21	0.59	−0.13	0.76
Category	Mean ± SD (ng/mL)	*p*	Mean ± SD (ng/mL)	*p*	Mean ± SD (pg/mL)	*p*
RF^−^	105.52 ± 39.58	0.44	674.47 ± 132.40	0.31	1.27 ± 0.34	0.94
RF^+^	73.97 ± 20.23		540.51 ± 117.69		1.17 ± 0.34	
ACPA^−^	86.65	0.74	502.00	0.64	0.87	0.28
ACPA^+^	76.77 ± 24.01		564.98 ± 129.34		1.20 ± 0.33	
UIP HRCT Pattern	83.81 ± 26.05	0.09	545.13 ± 120.36	0.99	1.19 ± 0.30	0.90
NSIP HRCT Pattern	65.51 ± 14.90		539.37 ± 135.50		1.17 ± 0.37	

ICAM-1: intercellular adhesion molecule 1; ET-1: endothelin 1; RA: rheumatoid arthritis; ILD: interstitial lung disease; CRP: C-reactive protein; ESR: erythrocyte sedimentation rate; FVC: forced vital capacity; FEV1: forced expiratory volume in one second; DLCO: diffusing capacity of the lung for carbon monoxide; SD: standard deviation; RF: rheumatoid factor; ACPA: anti-cyclic citrullinated peptide antibodies; UIP: usual interstitial pneumonia; HRCT: high-resolution computed tomography; NSIP: non-specific interstitial pneumonia. Significant results are highlighted in **bold**.

**Table 3:** Relationships of serum levels of E-selectin, ICAM-1, and ET-1 with clinical characteristics of SSc-ILD^+^ patients.

	E-Selectin Serum Levels		ICAM-1 Serum Levels		ET-1 Serum Levels	
Variable	*r*	*p*	*r*	*p*	*r*	*p*
Duration of SSc (years)	−0.19	0.45	−0.33	0.22	−0.02	0.96
CRP (mg/dL)	0.39	0.17	0.03	0.92	0.23	0.48
ESR (mm/1st hour)	0.12	0.69	0.15	0.64	0.47	0.12
FVC (% predicted)	**−0.64**	**<0.01**	−0.41	0.11	−0.41	0.12
FEV1 (% predicted)	**−0.56**	**0.02**	−0.37	0.16	−0.32	0.22
DLCO (% predicted)	**−0.56**	**0.02**	−0.41	0.11	−0.47	0.07
Category	Mean ± SD (ng/mL)	*p*	Mean ± SD (ng/mL)	*p*	Mean ± SD (pg/mL)	*p*
ANA^−^	65.21	0.76	534.00	0.36	0.82	0.83
ANA^+^	84.10 ± 37.35		511.91 ± 120.47		1.42 ± 0.83	
ACA^−^	85.39 ± 36.17	0.27	522.32 ± 113.70	0.19	1.37 ± 0.84	0.79
ACA^+^	40.69		357.00		1.66	
ATA^−^	68.46 ± 34.43	0.07	487.58 ± 118.87	0.17	1.32 ± 0.88	0.72
ATA^+^	97.84 ± 34.11		538.70 ± 116.11		1.45 ± 0.80	
UIP HRCT Pattern	99.69 ± 43.67	0.17	542.40 ± 130.37	0.10	1.42 ± 0.59	0.33
NSIP HRCT Pattern	80.93 ± 34.16		496.98 ± 113.98		1.40 ± 0.89	

ICAM-1: intercellular adhesion molecule 1; ET-1: endothelin 1; SSc: systemic sclerosis; ILD: interstitial lung disease; CRP: C-reactive protein; ESR: erythrocyte sedimentation rate; FVC: forced vital capacity; FEV1: forced expiratory volume in one second; DLCO: diffusing capacity of the lung for carbon monoxide; SD: standard deviation; ANA: antinuclear antibodies; ATA: anti-topoisomerase I antibodies; UIP: usual interstitial pneumonia; HRCT: high-resolution computed tomography; NSIP: non-specific interstitial pneumonia. Significant results are highlighted in **bold**.

**Table 4:** Characteristics of all the patients of the study.

	Study Objective Groups		Comparison Groups		
	RA-ILD^+^ n = 21	SSc-ILD^+^ n = 21	RA-ILD^−^ n = 25	SSc-ILD n = 20	IPF n = 21
Sex (women), n (%)	9 (45.9)	13 (61.9)	15 (60.0)	18 (90.0)	7 (33.3)
Age at study (years), mean ± SD	66.5 ± 10.1	60.3 ± 7.0	60.1 ± 11.8	56.6 ± 15.4	69.2 ± 10.0
Smoking ever, n (%)	13 (65.0)	11 (52.4)	13 (52.0)	11 (55.0)	16 (76.2)
AD duration (years), mean ± SD	9.2 ± 10.2	10.8 ± 8.3	4.1 ± 7.4	9.6 ± 8.1	-
CRP (mg/dL), mean ± SD	1.1 ± 1.1	0.7 ± 1.4	0.5 ± 0.5	0.5 ± 0.5	-
ESR (mm/1st hour), mean ± SD	22.8 ± 27.2	20.1 ± 15.9	14.4 ± 12.4	17.2 ± 13.4	-
**Antibody status**					
RF^+^, n (%)	17 (81.0)	-	11 (44.0)	-	-
ACPA^+^, n (%)	19 (90.4)	-	15 (60.0)	-	-
ANA^+^, n (%)	-	19 (95.0)	-	18 (90.0)	-
ACA^+^, n (%)	-	1 (5.0)	-	9 (45.0)	-
ATA (anti-Scl70) ^+^, n (%)	-	10 (50.0)	-	4 (20.0)	-
**Pulmonary function tests**					
FVC (% predicted), mean ± SD	95.2 ± 24.1	88.4 ± 27.1	99.2 ± 16.0	106.6 ± 15.9	84.9 ± 14.7
FEV1 (% predicted), mean ± SD	92.2 ± 21.0	87.3 ± 25.6	94.9 ± 22.0	101.9 ± 17.8	87.3 ± 19.6
DLCO (% predicted), mean ± SD	43.3 ± 15.9	47.5 ± 19.5	79.9 ± 20.0	71.5 ± 15.3	43.6 ± 18.4
**HRCT**					
Pulmonary involvement in HRCT, n (%)	21 (100.0)	21 (100.0)	0 (0.0)	0 (0.0)	21 (100.0)
UIP pattern, n (%)	11 (52.4)	3 (14.3)	-	-	21 (100.0)
Probable UIP pattern, n (%)	2 (9.5)	3 (14.3)	-	-	0 (0.0)
NSIP pattern, n (%)	7 (33.3)	14 (66.7)	-	-	0 (0.0)
Non-NSIP pattern, n (%)	1 (4.8)	1 (4.7)	-	-	0 (0.0)

RA: rheumatoid arthritis; ILD: interstitial lung disease; SSc: systemic sclerosis; IPF: idiopathic pulmonary fibrosis; SD: standard deviation; AD: autoimmune diseases; CRP: C-reactive protein; ESR: erythrocyte sedimentation rate; RF: rheumatoid factor; ACPA: anti-cyclic citrullinated peptide antibodies; ANA: antinuclear antibodies; ACA: anti-centromere antibodies; ATA: anti-topoisomerase I antibodies; FVC: forced vital capacity; FEV1: forced expiratory volume in one second; DLCO: diffusing capacity of the lung for carbon monoxide; HRCT: high-resolution computed tomography; UIP: usual interstitial pneumonia; NSIP: non-specific interstitial pneumonia.

## Data Availability

All data generated or analyzed during this study are included in this published article and in the Supplementary Material.

## Supplementary Materials

The supporting information can be downloaded at: https://www.mdpi.com/article/10.3390/ijms241512518/s1.
