# Supplementary material for: E-Selectin, ICAM-1, and ET-1 Biomarkers Address the Concern of the Challenging Diagnosis of Interstitial Lung Disease in Patients with Autoimmune Diseases
Source: Int J Mol Sci. 2023 Aug 7;24(15):12518. doi: 10.3390/ijms241512518 (PMC10420063; doi:10.3390/ijms241512518)
Supplement: Supplementary file 1 [file ijms-24-12518-s001.zip › ijms-2536674-supplementary.pdf]

E-selectin, ICAM-1, and ET-1 biomarkers address the concern of the challenging diagnosis of interstitial lung disease in patients with autoimmune diseases

Verónica Pulito-Cueto[1,2*¥], Sara Remuzgo-Martínez[3*], Fernanda Genre[3*], Belén Atienza-Mateo[1,2], Víctor M. Mora-Cuesta[1,4], David Iturbe-Fernández[1,4], Leticia Lera-Gómez[5], María Sebastián Mora-Gil[1,2], Virginia Portilla[1,2], Alfonso Corrales[1,2], Ricardo Blanco[1,2], José M. Cifrián[1,4,6], Miguel A. González-Gay[3,6,7] and Raquel López-Mejías[1,2**]**

Additional file S1

Table S1. Serum levels of E-selectin, ICAM-1, and ET-1 in all individuals of the study.

	E-selectin serum levels (ng/mL) *(Mean ± SD)*	***ICAM-1 serum levels (ng/mL)*** *(Mean ± SD)*	***ET-1 serum levels (pg/mL)*** *(Mean ± SD)*
**AD-ILD^{+}**	81.42 ± 30.65	532.60 ± 121.90	1.27 ± 0.61
**RA-ILD^{+}**	77.55 ± 22.85	556.40 ± 125.40	1.17 ± 0.33
**SSc-ILD^{+}**	85.11 ± 36.79	507.70 ± 116.20	1.37 ± 0.80
**AD-ILD^{-}**	60.72 ± 19.07	439.0 ± 77.03	0.99 ± 1.09
**RA-ILD^{-}**	57.27 ± 14.55	465.40 ± 80.55	0.86 ± 0.21
**SSc-ILD^{-}**	64.87 ± 23.09	404.30 ± 57.30	1.14 ± 0.39
**IPF**	76.75 ± 20.81	527.40 ± 75.48	1.09 ± 0.38

ICAM-1: intercellular adhesion molecule 1; ET-1: endothelin 1; SD: standard deviation; AD: autoimmune disease; ILD: interstitial lung disease; RA: rheumatoid arthritis; SSc: systemic sclerosis; IPF: idiopathic pulmonary fibrosis.
